# Systematic analysis of nonfatal suicide attempts and further diagnostic of secondary injury in strangulation survivors: A retrospective cross‐sectional study

**DOI:** 10.1002/hsr2.1572

**Published:** 2023-10-02

**Authors:** Thorleif Etgen, Manuel Stigloher, Hans Förstl, Peter Zwanzger, Michael Rentrop

**Affiliations:** ^1^ Klinik und Poliklinik für Psychiatrie und Psychotherapie Technische Universität München München Germany; ^2^ Klinik für Neurologie, Klinikum Traunstein Germany; ^3^ Institut für Geschichte und Ethik der Medizin Technische Universität München München Germany; ^4^ Department of Psychiatry Ludwig Maximilian University München Germany; ^5^ kbo‐Inn‐Salzach‐Klinikum, Klinik für Psychiatrie, Psychotherapie und Psychosomatik Wasserburg am Inn Germany

**Keywords:** dissection, neuroimaging, nonfatal suicide attempt, strangulation, suicide

## Abstract

**Background and Aims:**

Data on nonfatal suicide attempts in Germany are sparse. The study aimed to analyze data on nonfatal suicide attempts and consecutive diagnostic steps to identify secondary injuries after strangulation.

**Methods:**

All admissions after nonfatal suicide attempt in a large Bavarian psychiatric hospital between 2014 and 2018 were reviewed and the methods were analyzed.

**Results:**

A total of 2125 verified cases out of 2801 registered cases of nonfatal suicide attempts were included in further analysis. The most common methods were intoxication (*n* = 1101, 51.8%), cutting (*n* = 461, 21.7%), and strangulation (*n* = 183, 8.6%). Among survivors of strangulation with external neck compression (*n* = 99, 54.1%), no diagnostic steps were performed in 36 (36.4%) patients and insufficient imaging in 13 (20.6%) patients. Carotid artery dissection was detected in two (4.0%) of 50 patients with adequate neuroimaging.

**Conclusions:**

This study provides details on nonfatal suicide attempts in Germany. Slightly more than half of the patients with strangulation underwent adequate diagnostic work‐up, with 4.0% being diagnosed with dissection. Further studies with systematic screening for dissection after strangulation in psychiatric hospitals are recommended to reduce possible under‐reporting.

## 1 INTRODUCTION

Suicide statistics represent an important health indicator for any country, but suicide rates are often underestimated.^1^ Strangulation was the most common method of suicide in Germany in 2021, accounting for 43.9% of suicides.^2^ Data on methods and circumstances of nonfatal suicide attempts are even more sparse^3^ although secondary prevention and further diagnostic of secondary injury are of utmost importance. Especially in near‐hanging or nonfatal strangulation, a dissection of the cranial arteries is a rare but severe complication leading to a secondary disabling or fatal stroke.^4^ Only a few individual case descriptions and some studies from emergency medicine from the last 15 years have yet covered this topic with heterogeneous results between study centers resulting mainly from different search strategies and various imaging procedures.^5^, ^6^, ^7^, ^8^, ^9^, ^10^ A systematic evaluation of larger data sets from admissions to a psychiatric hospital has not yet been reported.

This study (a) analyses basic data of nonfatal suicide attempts, (b) categorizes different methods of nonfatal suicide attempts, (c) assesses the diagnostic process in patients with near‐hanging or survived strangulation, and (d) evaluates the incidence of dissection of the cranial arteries after near‐hanging or survived strangulation by analyzing the literature of the last 35 years. Based on the findings of this study, future recommendations will be developed.

## 2 METHOD

Patient data of the kbo‐Inn‐Salzach‐Klinikum Wasserburg am Inn in Germany from 2014 to 2018 were analyzed. The kbo‐Inn‐Salzach‐Klinikum Wasserburg am Inn is a large specialist hospital for psychiatry, psychotherapy, psychosomatics, neurology, and geriatrics. Its catchment area covers the entire south‐eastern Upper Bavarian region with over 900,000 inhabitants.

### 2.1 Basic documentation (BADO) form

Over the past decades, there have been many discussions regarding quality assurance and uniform documentation in psychiatry. Since 1995, the “basic documentation form” (BADO form) of the German Society for Psychiatry and Psychotherapy, Psychosomatics and Neurology (DGPPN) has been a standardized way for psychiatric hospitals to record data on patients systematically and to evaluate them uniformly, for example, for monitoring the success of treatment^11^ and predicting inpatient suicides and nonfatal suicide attempts.^12^ In the BADO form, the attending physician documents important parameters about the patient on admission and discharge. In total, the document comprises more than 70 items that are routinely collected.^13^ In addition to personal and sociological data such as gender, age, living situation and occupation, data on the diagnosis, the course of the disease, and the admission situation are also recorded. Among other things, there is the option of marking a checkbox for “suicide attempt” under the medical history data. The BADO admission forms marked in this way serve as the basis for the data set of this study.

### 2.2 Inclusion criteria

All inpatients with documentation in the BADO as “certainly dangerous suicide attempt,” “dangerous suicide attempt” or “other suicide attempt” in 2014–2018 period were included. All these cases were examined thoroughly based on the medical records to verify the BADO registration as a nonfatal suicide attempt. After the exclusion of nonverified suicide attempts, all remaining cases of nonfatal suicide attempts were further divided into different categories.

### 2.3 Exclusion criteria

Duplicate cases or the cases without any medical details were excluded. In addition, the cases of suicide gestures were excluded. Suicide gestures were defined by the intent to die criterion and encompassed those who self‐injured not to die but to communicate with others.^14^

### 2.4 Categories

To enable a uniform evaluation of the heterogeneous cases, a modified categorization used by the German Federal Statistical Office (“Statistisches Bundesamt”) was applied and 14 categories of nonfatal suicide attempts were defined.^2^ Our aim was to define as few categories as necessary to maintain clarity, but still enough to assign all nonfatal suicide attempts to one category clearly. For example, suicides due to intoxication are classified by the German Federal Statistical Office into the following categories: (a) antiepileptic drugs, hypnotics, antiparkinsonian drug(s) and psychotropic substance(s); (b) narcotics and psychodysleptics, etc.; (c) nonopioid analgesic(s), antipyretic(s) and antirheumatic(s); (d) other medicines, drugs and biologically active substance(s); (e) alcohol. Our modified categorization of nonfatal suicide attempts summarized the categories (a)–(e) into one category, “intoxication.”^2^ All following methods of nonfatal suicide attempts were sorted according to the patient's intention, regardless of the quality of the execution:

- Freezing (nonfatal suicide attempt by intentionally lowering the body temperature).
- Shooting (nonfatal suicide attempt by gunshot which comprises cases of gun violence directed against oneself and requests directed at other persons to be shot).
- Suffocation (nonfatal suicide attempt through lack of oxygen).
- Drowning.
- Intoxication (nonfatal suicide attempt by poisoning or overdosing).
- Cutting (nonfatal suicide attempt by blood loss through stab or cut wounds).
- Jumping (nonfatal suicide attempt by trauma and all cases in which patients deliberately threw themselves from a greater height or attempted to do so).
- Road traffic accident (a case in which patients attempted to cause a traffic accident or be killed by other road users).
- Strangulation (defined as external neck compression that may lead to fatal injury to vital structures in the neck such as the airways, blood vessels and nerves^15^ and can occur in many ways including hanging which is caused by the pull of the body's weight).
- Electrocution (attempted killing by electricity).
- Blunt force trauma.
- Burning (a case in which patients attempted to set themselves or their surroundings on fire to die from the burns or high temperatures; carbon dioxide poisoning is dealt with in the category “suffocation”).
- Ingestion of objects (swallowing of (nontoxic) objects to cause harm to themselves).
- Refusal to eat (cases in which death was to be caused by deliberately stopping the intake of food or fluids, resulting in starvation, or dying of thirst).

Classification into these categories was carried out by two researchers blinded from each other and inter‐rater reliability was calculated. In any remaining differences, a common decision had to be reached by consulting a third researcher.

Finally, in all cases of the category “strangulation” admission details were thoroughly checked to exclude those cases in which no external neck compression was documented. Among the remaining cases, we analyzed if and which diagnostic procedure was performed to search for any secondary injury, especially a dissection of the cranial arteries. Only ultrasound, computer tomography (CT) with angiography and magnetic resonance imaging (MRI) with angiography or digital subtraction angiography were considered adequate for the detection of a dissection.^16^ Based on these results, the risk of suffering a dissection of the cranial arteries because of nonfatal strangulation with suicidal intent was estimated.

## 3 RESULTS

**Figure 1:**
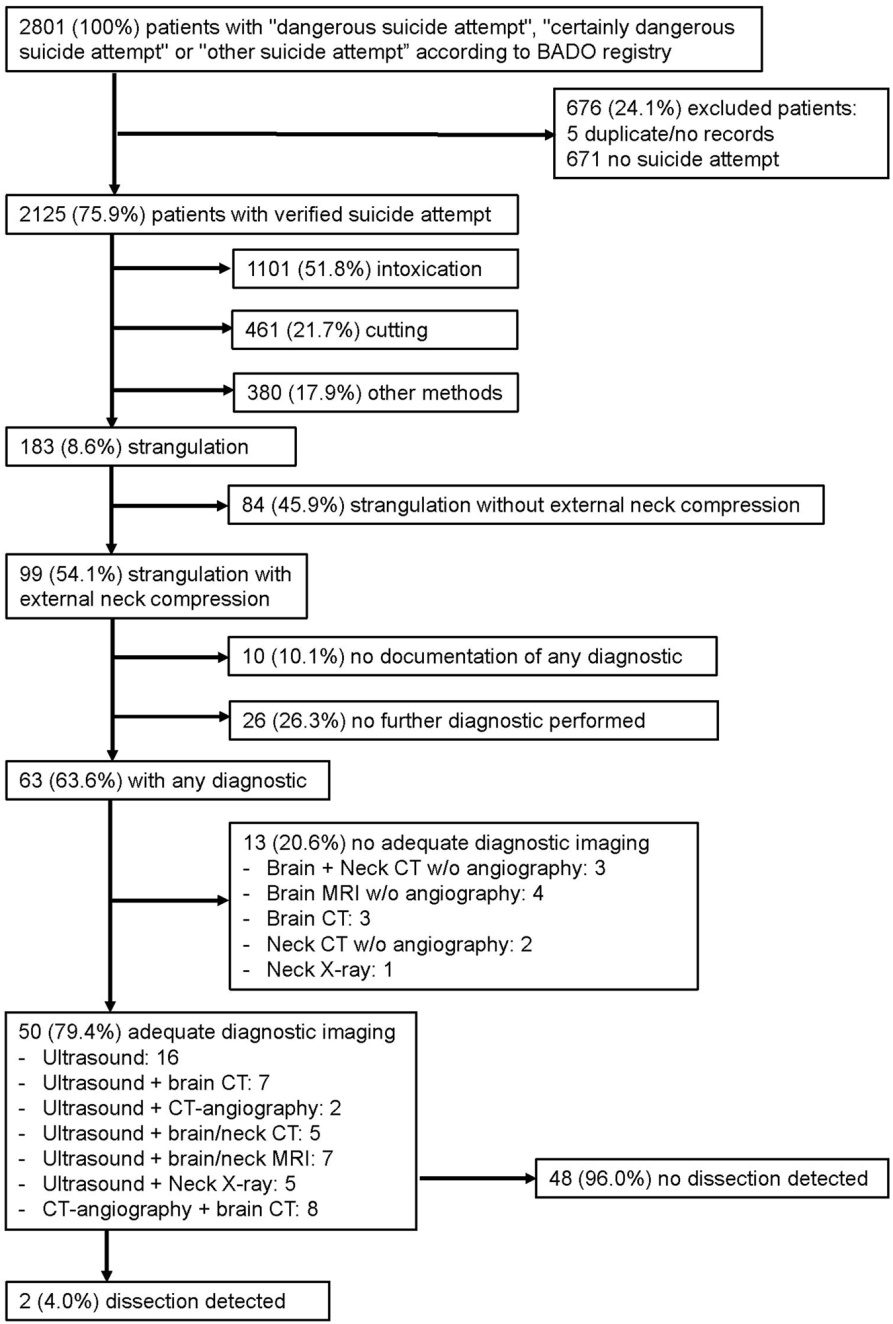
Flowchart of patients.

In 2014–2018 period, a total of 2801 cases of “dangerous suicide attempt,” “certainly dangerous suicide attempt” or “other suicide attempt” were documented at the kbo‐Inn‐Salzach‐Klinikum. Of these, five cases (0.3%) were either duplicated or no medical record was available. After examination of the medical records, further 671 cases (24.0%) of suicide gestures were excluded. A total of 676 cases (24.1%) were therefore excluded. A total of 2125 cases (75.9%) remained for further analysis (Figure 1).

### 3.1 Age distribution

A total of 1163 (54.7%) nonfatal suicide attempts were made by women and 962 (45.3%) by men. Among men, between 21 and 25 years of age was the most frequently represented cohort, however, among women the most frequently represented age was between 16 and 20 years. In both groups, a second peak is noticeable between 51 and 55 years which is much more pronounced among women.

### 3.2 Method distribution

The most frequent method used in more than half of the nonfatal suicide attempts was intoxication (*n* = 1101, 51.8%), followed by cutting (*n* = 461, 21.7%) and strangulation (*n* = 183, 8.6%). The distribution of the remaining methods is shown in Table 1. Inter‐rater reliability was high (Kappa = 0.929, 95% confidence interval: 0.918–0.941).

**Table 1:** Distribution of methods of nonfatal suicide attempts according to BADO.

Method of nonfatal suicide attempt	Number of patients (Percentage)	Number of male/female patients
Intoxication	1101 (51.8)	423/678
Cutting	461 (21.7)	220/241
Strangulation	183 (8.6)	111/72
Jumping	108 (5.1)	58/50
Traffic accident	103 (4.8)	61/42
Drowning	46 (2.2)	20/26
Suffocation	40 (1.9)	28/12
Swallowing objects	26 (1.2)	8/18
Refusal to eat	16 (0.8)	7/9
Electrocution	12 (0.6)	4/8
Blunt force trauma	11 (0.5)	8/3
Shooting	8 (0.4)	8/0
Burning	6 (0.3)	5/1
Freezing to death	4 (0.2)	1/3

### 3.3 Strangulation

The category “strangulation” contained 111 male and 72 female patients. The age distribution is comparable to that of all nonfatal suicide attempts, with both sexes having a peak in the group of 26–30 years. Among men, a second peak appears between 66 and 70 years old and among women in the group of 56–60 years.

### 3.4 Diagnostic steps after strangulation

This category comprised 99 patients with documented external neck compression; in the remaining 84 patients either no external neck compression occurred or could not be clearly verified (Figure 1). In these 99 patients after strangulation with documented external neck compression, further diagnostic steps were performed in 63 (63.6%) patients. Adequate diagnostic imaging was performed in 50 patients and revealed a dissection in two patients (4.0%).

After strangulation, a 46‐year‐old woman presented with a mild difference in pupillary diameter size and diagnostic (ultrasound and MRI) revealed a dissection of the left common carotid artery. An 88‐year‐old man had no clinical symptoms after near‐hanging, but CT‐angiography as part of trauma screening showed dissection of the right distal common carotid artery at the level of the larynx. In both cases, therapy with acetyl salicylic acid was started and the subsequent clinical course was uneventful.

## 4 DISCUSSION

**Figure 2:**
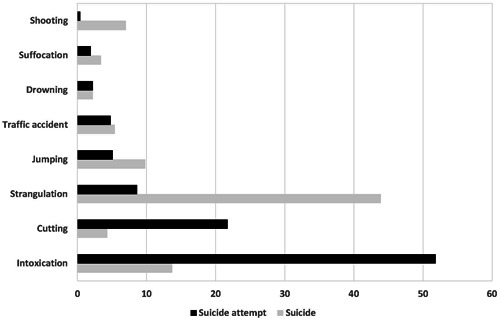
Most common categories (%) of suicides in Germany in 2021 and nonfatal suicide attempts in our study.

This study provides detailed data on different methods of nonfatal suicide attempts in Germany. Strangulation constitutes the most frequent method in nearly half of all suicides (43.9%) in Germany in 2021,^2^ whereas in nonfatal suicide attempts, strangulation is used in less than 10% of all cases. Hanging has been the most common suicide method in Germany for many years, and it is also one of the most common suicide methods worldwide.^17^ Moreover, hanging is the second lethal method with a case fatality rate of 84.5%.^18^ In contrast, intoxication is found in more than half of all nonfatal suicide attempts but only in 13.7% of all suicides (Figure 2).^2^

These data might help in further prevention of suicide as nonfatal suicide attempts remain the strongest risk factor for future suicide and nonfatal suicide attempts appear to be increasing but the use of support and services among those who survived a nonfatal suicide attempt has not increased.^19^ Though case fatality following a nonfatal suicide attempt by hanging is around 70%, the majority (80%–90%) of those who reach the hospital alive survive.^20^ Restricted access to lethal means like firearms, drugs, domestic gas, and car exhaust with high content of carbon monoxide is associated with a decline in suicide^21^ pointing to successful intervention based on analysis of nonfatal suicide attempts.

This study systematically assesses the diagnostic process in patients with near‐hanging or nonfatal strangulation and evaluates the incidence of dissection of cranial arteries in a large psychiatric setting. The results show that in one‐third of all patients after strangulation no further diagnostic steps of a possible dissection of cranial arteries were performed. According to our results, the incidence of dissection of cranial arteries in patients after strangulation in a nonfatal suicide attempt is 4% though a possible underreporting may exist as no diagnostic steps at all were performed in one‐third and inadequate neuroimaging in one‐fifth of these patients.

All previous studies have been thoroughly reviewed and revealed several differences (Table 2).^5^, ^6^, ^7^, ^8^, ^9^, ^10^

**Table 2:** Overview of studies with diagnostic imaging in strangulation patients.

**First author**	**Ribaute** ^9^	**Schuberg** ^6^	**Zuberi** ^7^	**Matusz** ^8^	**Nichols** ^10^	**Schellenberg** ^5^	**Etgen et al. (this study)**
**Admission unit**	Emergency department or intensive care unit	Level I Trauma center	Emergency department	Level I trauma center	Level I trauma center	Trauma and Surgical Critical Care	Psychiatry hospital
**Period**	2010–2017	2008–2014	2009–2016	2008–(2019)	1990–2007	2008–2015	2014–2018
**Search criteria**	Keyword “hanging” in CT reports	Clinical impression of hanging, strangulation, or asphyxiation (E93.0)	CT‐angiography of the neck with indication “strangulation” or similar word trunks	Asphyxiation injury in 3 databases	E‐code hanging (E953.0, E983.0)	E‐code hanging (E913.8, E953.0, E983.0)	Nonfatal suicide attempt by strangulation or hanging
**Imaging**	CT angiography	CT angiography	CT angiography	CT or MR angiography	ultrasound, angiogram, CT angiogram	CT angiography	Ultrasound, CT or MR angiography
**Number of screened patients**	242	100	Not given	349	67	71	183
**Number of analyzed patients with adequate imaging (%)**	162 (66.9%)	66 (66.0%)	142	208 (59.6%)	32 (47.8%)	69 (97.2%)	42 (23.0%)
**Number of patients with dissection (%)**	4 (2.5%)	0	3 (2.1%)	2 (0.6%)	2 (6.3%)	3 (4.3%)	2 (4.8%)

First, all previous studies were exclusively performed at trauma centers or emergency departments. However, in Germany, patients are admitted directly to a psychiatric hospital after a nonfatal suicide attempt if no serious injuries are apparent or no special observation is required.

Second, the search strategy for patients varied from a radiologic report‐based selection^7^, ^9^ to medical records with a clinical diagnosis based on the “International Classification of Disease” (ICD 9).^5^, ^6^, ^10^ On the one hand, the radiologic report‐based selection excluded all patients with strangulation who did not receive any imaging.^7^, ^9^ On the other hand, the ICD‐based selection included only near‐hanging victims (E983.0) but patients after strangulation or suffocation by other specified (E983.8) or by unspecified means (E983.9) were not included.^5^, ^6^, ^10^

Third, most studies relied solely on CT‐angiography^5^, ^6^, ^7^, ^9^ excluding all patients with any other diagnostic imaging. Only two studies used other diagnostic imaging like MR angiography^8^ or ultrasound.^10^

Fourth, the portion of performed diagnostic imaging among in most studies ranged from 47.8% to 66.9%,^6^, ^8^, ^9^, ^10^ only one study managed to investigate nearly all screened patients.^5^ Therefore, the validity of imaging results is limited if in most of these studies, a substantial minority of patients was excluded due to absent imaging.

Among 721 patients from all these studies with survived strangulation and adequate imaging, 16 (2.2%) cases of dissection of the cranial arteries were detected which is lower than in our current analysis.

The numerous differences of these studies consist of the type of study center (emergency department, psychiatry hospital), the search strategy with heterogenous inclusion and exclusion criteria (radiologic report‐based selection, ICD‐based selection), the various application of imaging procedures (CT‐/MR‐angiography, ultrasound) and the proportion of examined patients. This explains the divergent results and conflicting recommendations of further diagnostic to detect secondary injury, including dissection of the cranial arteries. Some studies question systemic CT angiography^6^, ^7^, ^9^ whereas other studies advocate its application.^5^, ^10^ On the one hand, CT angiography is cost‐intensive, carries the risk of contrast agent reaction and uses potentially harmful X‐ray limiting its use as a screening procedure.^22^ On the other hand, overlooking a secondary injury after a nonfatal suicide attempt, such as a dissection of cranial arteries, carries the risk of a subsequent disabling or fatal stroke. As up to 40% of fatal strangulations have no external signs, and most of surviving victims have few or minor injuries, the selection of patients for further imaging based only on clinical grounds seems unreliable.^15^

Limitations of our study encompass the single‐center design, which may not reflect the nonfatal suicide attempt rate in Germany and the rate of performed diagnostic imaging after strangulation.

## 5 CONCLUSION

Further studies with systematic screening for dissection after strangulation in psychiatric hospitals are recommended. We suggest establishing a multicentre prospective register; collecting basic clinical data (method and details of strangulation, presence of clinical injury, neurological examination, pain assessment, etc.); performing ultrasound as a screening procedure for dissection in all cases of strangulation survivors and adding CT or MR angiography (depending on local expertise) in any doubtful cases. This procedure would allow to develop a step‐by‐step assessment protocol in strangulation survivors and could help reduce secondary injury after a nonfatal suicide attempt.

## AUTHOR CONTRIBUTIONS

**Thorleif Etgen**: Conceptualization; formal analysis; investigation; project administration; writing—original draft. **Manuel Stigloher**: Conceptualization; formal analysis; investigation; writing—review & editing. **Hans Förstl**: Conceptualization; writing—review & editing. **Peter Zwanzger**: Conceptualization; writing—review & editing. **Michael Rentrop**: Conceptualization; formal analysis; investigation; writing—review & editing.

## CONFLICT OF INTEREST STATEMENT

All authors declare no conflict of interest.

## ETHICS STATEMENT

The ethics committee of the Technische Universität München approved the study (2022‐392‐S‐NP). The authors declare that the current version of the Helsinki Declaration has been observed. Being a retrospective study, an exemption from requiring an informed consent was granted by the ethics committee of the Technische Universität München.

## TRANSPARENCY STATEMENT

The lead author Thorleif Etgen affirms that this manuscript is an honest, accurate, and transparent account of the study being reported; that no important aspects of the study have been omitted; and that any discrepancies from the study as planned (and, if relevant, registered) have been explained.

## Data Availability

All relevant data are reported within the paper and are available from the corresponding author upon reasonable request. All authors have read and approved the final version of the manuscript. The corresponding author had full access to all of the data in this study and takes complete responsibility for the integrity of the data and the accuracy of the data analysis.
